# PManalyzer: A Software Facilitating the Study of Sensorimotor Control of Whole-Body Movements

**DOI:** 10.3389/fninf.2019.00024

**Published:** 2019-04-05

**Authors:** Thomas H. Haid, Matteo Zago, Arunee Promsri, Aude-Clémence M. Doix, Peter A. Federolf

**Affiliations:** ^1^Department of Sport Science, University of Innsbruck, Innsbruck, Austria; ^2^Department of Electronics, Information and Bioengineering, Politecnico di Milano, Milan, Italy; ^3^Department of Physical Therapy, University of Phayao, Mae Ka, Thailand

**Keywords:** sensorimotor control, motion analysis, clinical gait analysis, postural control, coordination, principal component analysis PCA

## Abstract

Motion analysis is used to study the functionality or dysfunctionality of the neuromuscular system, as human movements are the direct outcome of neuromuscular control. However, motion analysis often relies on measures that quantify simplified aspects of a motion, such as specific joint angles, despite the well-known complexity of segment interactions. In contrast, analyzing whole-body movement patterns may offer a new understanding of movement coordination and movement performance. Clinical research and sports technique evaluations suggest that principal component analysis (PCA) provides novel and valuable insights into control aspects of the neuromuscular system and how they relate to coordinative patterns. However, the implementation of PCA computations are time consuming, and require mathematical knowledge and programming skills, drastically limiting its application in current research. Therefore, the aim of this study is to present the Matlab software tool “PManalyzer” to facilitate and encourage the application of state-of-the-art PCA concepts in human movement science. The generalized PCA concepts implemented in the PManalyzer allow users to apply a variety of marker set independent PCA-variables on any kinematic data and to visualize the results with customizable plots. In addition, the extracted movement patterns can be explored with video options that may help testing hypotheses related to the interplay of segments. Furthermore, the software can be easily modified and adapted to any specific application.

## Introduction

Sensorimotor control of movements is one of the most important functions of the nervous system. It involves *detecting* the physical state which the biomechanical system is in; *processing* this information to determine which changes to the system are desired or need to be opposed; and *activating* the motor system to generate the forces that produce the required changes to the system. From a biophysical viewpoint, the *state* of the biomechanical system is fully described, when the position and velocity of the body segments are known. Thus, full-body motion analysis offers an approach for studying the function of the nervous system by determining, on the one hand, the state of the system and thus the input to the various sensory systems, and, on the other hand, the accelerations of the body segments and thus the resultant output of the neuromuscular system.

However, multi-segment human movements allow many degrees of freedom DOF and typically allow a large variety of different movement strategies to successfully achieve a goal (Bernstein, 1967), i.e., human movements are mechanically complex. Therefore, conventional movement analyses often look into specific, pre-determined aspects of a motion. Such analyses often neglect important information about segment interactions; and the complex nature of these interactions makes a priori variable determination prone to false identification of important aspects. That is why other approaches quantify whole body kinematics (Honegger et al., 2013; Boström et al., 2018). Nevertheless, most of these approaches still rely on pre-defined aspects of specific movements such as angles, torques, or segment trajectories.

In the past two decades several principal component analysis (PCA) based approaches were developed for various applications in kinematic data analysis (Sadeghi et al., 1997; Troje, 2002; Daffertshofer et al., 2004; Wang et al., 2014), with the aim of determining relevant aspects of a motion in an unbiased and data driven way. One of these approaches identifies whole-body movement components (Troje, 2002; Daffertshofer et al., 2004), later called principal movements PM_k_ (Federolf et al., 2012), thus reducing data complexity without neglecting segment interactions. In this framework, a PCA yields eigenvectors ***PC***_***k***_, eigenvalues *EV*_*k*_ and score time-series called principal positions ***PP***_***k***_(*t*). Each ***PC***_***k***_ defines one type of movement or movement strategy that the respective PM_k_ describes, while each *EV*_*k*_ determines the amount of total variance in the data explained by the respective PM_k_. Furthermore, the scores ***PP***_***k***_(*t*) determine the evolution of the respective PM_k_ over time.

Among the first papers applying PCA in this sense were studies on walking patterns and gait forms (Troje, 2002; Daffertshofer et al., 2004; Verrel et al., 2009). In these studies, a separate PCA was conducted for each trial and the individual EV-spectra characterizing the amount of contribution of each individual postural strategy were compared. On the one hand, this approach allowed programming a motion synthesizer that displays gait forms according to different classifiers such as gender, weight, and emotional condition (Troje, 2002). On the other hand, it could be shown that gait regularity is not only affected by cognitive dual-tasking, but that different age groups display different changes in regularity (Verrel et al., 2009).

These results established PCA as a useful tool to analyze human movements. However, only EV-spectra describing the contribution of trial specific movement patterns could be compared, thus the comparison of movements between subjects or trials remained unsolved. Soon after, it was shown that one PCA could be conducted on several trials of various participants simultaneously, if the datasets were normalizing appropriately (Federolf P. et al., 2013). This approach enabled the comparison of the movement executions ***PP***_***k***_(*t*) between trials. Furthermore, the relative contribution of each PM_k_ to a trial's overall variance (corresponding to the *EV*_*k*_) was quantified with the variable *rVAR*_*k*_ computed on the ***PP***_***k***_(*t*).

Amongst others, the *rVAR*_*k*_ and ***PP***_***k***_(*t*) have provided new insights into the execution of sports techniques in alpine skiing, cross-country skiing, karate, dancing, cycling and race-walking (Donà et al., 2009; Moore et al., 2011; Masurelle et al., 2013; Federolf et al., 2014; Gløersen et al., 2017; Zago et al., 2017a). Moreover, related variables such as residual variances *RV*_*k*_ or relative standard deviations *rSTD*_*k*_ have been used to quantify the dimensionality of coordinative tasks such as juggling or balancing (Zago et al., 2017b; Haid and Federolf, 2018).

While the studies discussed so far applied the PCA method to compare movements, they have not calculated velocities or accelerations, and thus have not studied the control of movements. Only in 2016 it was suggested to differentiate the ***PP***_***k***_-time series to obtain principal velocities ***PV***_***k***_ and principal accelerations ***PA***_***k***_ (Federolf, 2016). Since then, ***PA***_***k***_ and variables based on ***PA***_***k***_ have been used to study differences in movement control due to aging (Haid et al., 2018) or leg dominance/laterality (Promsri et al., 2018a). The ***PP***_***k***_ and ***PP***_***k***_**-**variables were also applied in postural control research and linked to COP-time-series (Federolf, 2016), which are analyzed in a range of clinical applications that investigate impairments due to aging, overweight, back pain, concussion, multiple sclerosis, autism spectrum disorders, or Parkinson's disease (Fino et al., 2016; Huisinga et al., 2017; Lim et al., 2017; Yamagata et al., 2017; Han et al., 2018; MacRae et al., 2018; Nikaido et al., 2018). A recent study evaluated COP-irregularity by linking it to ***PP***_***k***_(*t*) irregularity and to the complexity of the movement structure as defined by *rSTD*_*k*_ (Haid et al., 2018).

Variables computed on PM time-series contain information about whole-body positioning, which allows studying the movements of the human body as a system, while preserving or possibly enhancing (Federolf P. A. et al., 2013) the ability to discriminate groups. Therefore, the PCA approach is well-suited for addressing any research questions where coordination or the interplay of segment movements is of importance. However, despite its research potential the implementation of PCA approaches requires a fair amount of programming and mathematical skills, and can be very time consuming. Therefore, the development of new PCA based variables and research output validation comparing different computational options is severely hampered.

The main goal of this paper is to present the PManalyzer-software. It generalizes many of the existing PCA concepts and was designed to motivate the development and validation of kinematic PCA related variables and methods within a user-friendly graphical environment. On the one hand, the software will allow researchers and clinicians without extensive programming or mathematical skills to perform PCA on kinematic data; on the other hand, it will allow users with more advanced knowledge in the area to adapt and further develop the software.

## Materials and Methods

The software was designed to pre-process the kinematic input data and then compute a PCA on it. Furthermore, the PManalyzer can compute a range of PCA variables. In this section the mathematical background of kinematic feature extraction and some of the most important variables are explained.

### General Data Model and Data Pre-processing

Typical kinematic data consists of 3D positions in time obtained by tracking the motion of *n* anatomical landmarks; either utilizing a motion capture system or video-tracking (Figueroa et al., 2003). The kinematic data is then available in matrix form in which the *N* = 3·*n* columns represent the time-series *s*_*i*_(*t*) (*i*∈{1, 2…, *N*}) of the respective x-y-z-coordinates of each anatomical landmark. Each row contains the measured 3D positions of all markers at one time-point:

$$D=\;\left[\begin{array}{ccc} s_{1}(t_{1}) & \cdots & s_{N}(t_{1})\\ \vdots & \ddots & \vdots \\ s_{1}(t_{T}) & \cdots & s_{N}(t_{T}) \end{array}\right],$$

Where *T* equals the number of measured time-points. The application of PCA to human movement is based on the idea of identifying linear whole-body movement patterns that dominate the recorded movements. However, when identifying movement patterns within a group of several subjects, both the mean positioning of a participant and anthropometrical differences distort the results. To reduce such distortions, the data of each subject can be centered, weighted and normalized (Federolf P. et al., 2013; Zago et al., 2017b; Haid et al., 2018).

The data is centered by subtracting the mean < ***s***_***i***_> of each individual time-series ***s***_***i***_ (each column) from the respective time-series $s_{i}^{c}=\;s_{i}-<s_{i}\;>$:

$$\begin{array}{l} D^{c}=\;\left[\begin{array}{ccc} s_{1}^{c}\left(t_{1}\right) & \cdots & s_{N}^{c}\left(t_{1}\right)\\ \vdots & \ddots & \vdots \\ s_{1}^{c}\left(t_{T}\right) & \cdots & s_{N}^{c}\left(t_{T}\right)\\ \; \end{array}\right], \end{array}$$

preventing differences in mean marker positioning in space to affect the results. Furthermore, a participant's weight distribution can influence marker movements. As an example, when moving a hand, less mass has to be accelerated and controlled, in comparison to moving a thigh. Therefore, each of the *N* time-series can be scaled according to the weight *w*_*i*_ (*i*∈{1, 2…, *N*}), that the respective marker represents:

$$\begin{array}{l} D^{c,\;w}=\;D^{c}\cdot \;\left[\begin{array}{ccc} w_{1} & \cdots & 0\\ \vdots & \ddots & \vdots \\ 0 & \cdots & w_{N}\\ \; \end{array}\right], \end{array}$$

Weighting has been applied in literature (Federolf P. et al., 2013; Gløersen et al., 2017; Haid et al., 2018; Promsri et al., 2018a), often considering gender-specific mass distributions (Defense Technical Information Center, 1988; de Leva, 1996; Gallagher and Heymsfield, 1998).

Another important aspect to be considered when comparing trials is that anthropometric differences can influence the amount of movement produced. Therefore, each data-set should be normalized according to application specific criteria:

$$\begin{array}{l} D^{c,\;w,n}=\;D^{c,w}\cdot \frac{1}{d_{norm}} \end{array}$$

Normalization factors *d*_*norm*_ such as the mean Euclidean distance (MED) (Federolf P. et al., 2013; Zago et al., 2017b) or the body height (Haid and Federolf, 2018; Haid et al., 2018) have been proposed to reduce anthropometric differences. In detail, the MED ensures that all subjects contribute equally to the overall variance, while the body height normalization scales the data to a trial-independent anthropometric parameter.

Once the data sets of each participant are centered, weighted and normalized^1^, one large data matrix ***D***^***all***^ is formed, containing all data sets of all *X* trials concatenated vertically (with index 1.X representing different subjects and/or several trials of different subjects):

$$\begin{array}{l} D^{all}=\;\left[\begin{array}{c} D_{1}^{c,\;w,n}\\ \vdots \\ D_{X\;}^{c,\;w,n} \end{array}\right] \end{array}$$

### Feature Extraction—PCA

After pre-processing, the eigenvectors ***PC***_***k***_and eigenvalues *EV*_*k*_ of the covariance matrix of ***D***^***all***^ are computed [implemented as SVD (Shlens, 2014)]. The eigenvectors ***PC***_***k***_ form a new orthonormal basis that spans posture space (Federolf P. et al., 2013), a space in which each of the axes determines one specific linear, one-dimensional whole-body movement. Furthermore, scores **S** can be obtained by projecting the data onto the new *PC*_*k*_ basis:

(1) $$\begin{array}{r} {S=D}^{all}\cdot PC_{k} \end{array}$$

The k-th column of the score matrix **S** can be interpreted as time-series ***PP***_***k***_**(***t***)** that quantitatively describes the evolution over time of the respective principal movement PM_k_, i.e., the manifestation of the one-dimensional PM_k_ defined by the corresponding ***PC***_***k***_:

$$\begin{array}{l} S=\;\left[\begin{array}{ccc} {PP}_{1}\left(t_{1}\right) & \cdots & {PP}_{N}\left(t_{1}\right)\\ \vdots & \ddots & \vdots \\ {PP}_{1}\left(t_{T}\right) & \cdots & {PP}_{N}\left(t_{T}\right) \end{array}\right] \end{array}$$

In addition, the eigenvalues *EV*_*k*_ describe the amount of variance (or movement) explained by each PM_k_ and are typically presented as percentages or relative eigenvalues *rEV*_*k*_^2^.

To compute one PCA on each trial separately can be done by running the software for each trial separately. However, this feature is not explicitly supported, because the authors recommend only comparing trials with respect to one PCA basis that describes the group as a whole. The benefit of the current procedure—being able to compare the ***PP***_***k***_**(***t***)** of trials—outweighs the benefit of obtaining several, trial-specific PCA-bases that only allow comparisons of *rEV*_*k*_-spectra, but not of ***PP***_***k***_(*t*). Moreover, the ***PP***_***k***_**(***t***)** can be used to compute variables that describe the subjects specific variance explained by each PM_k_ and further variables that quantify the additional aspects of a movement or of neuromuscular control.

### Interpretation of the Movement Components

As mentioned in the previous section, the ***PC***_***k***_ form a basis of the posture space. Moreover, they have the property that they point in the direction of the largest correlated variance expressed in the data. Therefore, they point in the direction of the most common patterns of correlated marker movements. As a consequence, the PM_k_ are *linear* approximations of the analyzed movements and interpreting them as *real* movements should be done with caution. For example, to explain non-linear movements such as rotations at least two linear components are needed. Nevertheless, for movements with small perturbations such as static balancing tasks (tandem, bipedal, one-legged) past research has found the PM_k_ to describe the main dynamics of well established, nonlinear movement strategies, e.g., ankle sway or upper body rotation (Haid and Federolf, 2018; Haid et al., 2018; Promsri et al., 2018a). Moreover, also for movements with higher amplitudes the PM_k_ have been found to reflect the main the dynamics of established movement strategies, such as isolated leg or arm swinging, trunk leaning, or coordinated multi-segment movements (Troje, 2002; Verrel et al., 2009; Eskofier et al., 2013; Gløersen et al., 2017; Zago et al., 2017a,b).

Advantages of analyzing the movement with PMs are that few variables are needed to approximate the movement to great detail and obtaining the PMs is data-driven—not postulated from subjective observations. Moreover, the PMs can be visualized which improves interpretation of results. In the current paper we further propose that movement analysis involving rotational movements of large amplitudes could additionally benefit from non-linear coordinate transformations. To the best of our knowledge, there is no literature to support this assumption, therefore, a motivational example will be presented.

### PCA Variables

In the following some of the most common kinematic PCA variables in literature are described. These, amongst others, are pre-implemented in the software.

#### Trial Specific Movement Structure or Composition

The *rEV*_*k*_ determine the overall variance explained by each PM_k_ either in the respective trial—if one PCA is computed for one trial—or in the overall variance produced by all trials—if one PCA is computed for the concatenated trials-matrix. In the latter, trial-specific relative variances *rVAR*_*k*_ can be computed that represent the explained variance of each PM_k_ (Federolf P. et al., 2013), analogously to the rEV_k_ for applications in which one PCA is computed for each trial. Therefore, the sum of all variances of each trial's individual ***PP***_***k***_(*t*) ~ time-series

$$\begin{array}{l} totVAR^{trial}:=\;\sum_{k}{\text{VAR}}_{\text{k}}^{\text{trial}}:=\sum_{k}\text{VAR}\left(PP_{k}^{trial}\left(\text{t}\right)\;\right), \end{array}$$

can be computed. The subject specific relative variances are then defined by

$$rVAR_{k}^{trial}=\frac{{\text{VAR}}_{\text{k}}^{\text{trial}}}{totVAR^{trial}}\;\cdot \;100.$$

To obtain a similar variable that quantifies the movement structure and explains the relative contributions to a movement, but scales as the original movement, the variance in the rVAR_k_ computation can be replaced by the standard deviation to compute trial-specific relative standard deviations rSTD_k_ (Haid and Federolf, 2018; Haid et al., 2018).

When the dimensionality of a movement is of interest, it makes sense to define subject specific cumulative relative variances as

$$CUM\_rVAR_{k}:\text{​ }=\frac{\Sigma_{1\leq \text{n}\leq k}{\text{VAR}}_{\text{n}}^{\text{trial}}}{totVAR^{trial}}\;\cdot \;100,$$

or analogously CUM_rSTD_k_, which explain the cumulative contribution of the respective component order. This can further be used to compute subject specific residual variances

$$RV^{3}:\text{ ​}=100-CUM\_rVAR_{m}=\frac{\sum_{k>m}^{}{\text{VAR}}_{\text{k}}^{\text{trial}}}{\sum_{k}^{}{\text{VAR}}_{\text{k}}^{\text{trial}}\;}\;\cdot \;100$$

where m is the highest PC-order included (Zago et al., 2017b,c).

#### Kinematics in Posture Space and Measures of Postural Control

Similarly to conventional kinematics in biomechanics (Federolf, 2016) the ***PP***_***k***_(*t*) time-series can be utilized to analyze the execution of movements with respect to their PM_k_. Different trajectories or performances can therefore be directly compared to another if the ***PP***_***k***_(*t*) of all trials are coordinates in the same posture space, i.e., if one only one PCA was computed.

Furthermore, the ***PP***_***k***_(*t*) can be utilized to compute principal velocities ***PV***_***k***_(*t*) and principal accelerations ***PA***_***k***_(*t*) by differentiating the ***PP***_***k***_ once and twice, respectively. The dynamics of all three PM time-series can be studied using conventional time-series analysis. For example, postural reconfiguration can be ascribed to acting external forces, such as gravity, and internal forces, such as acting muscle forces. Therefore, the ***PA***_***k***_(*t*) can be used to compute variables that characterize the neuromuscular control of movement, as they represent the acceleration of the postural movements. For example, it has been shown that the ***PA***_***k***_ can be used to quantify the amount and the variability of the neuromuscular control, by further defining variables *N*_*k*_ and σ_*k*_ (Haid et al., 2018; Promsri et al., 2018a), which represent the number of ***PA***_***k***_ -zero-crossings (changes in the direction in which the neuro-muscular control^4^ system influences the current motion) and the time-variability between the zero-crossings, respectively.

Table 1 contains a summary and a description of these PCA variables. However, any other type of time-series analysis that fits the research question may be applied to the three PM time-series.

**Table 1:** Summary and description of the variables.

**Abbreviation**	**Variable name**	**Description**
***PC***_***k***_	Principal components/eigenvectors	Contains the information about the marker movements that define the PM_k_
*EV*_*k*_	Eigenvalues	Absolute contribution of PM_k_ to overall variance
***PP***_***k***_(*t*)	Principal positions	Time-series that quantifies the evolution of the posture with respect to ***PC***_***k***_
***PV***_***k***_(*t*)	Principal velocities	Time-series that quantifies the velocity of the postural changes defined by PM_k_
***PA***_***k***_(*t*)	Principal accelerations	Time-series that quantifies the acceleration of the postural changes defined by PM_k_
*rEV*_*k*_	Relative eigenvalues	Relative contribution of PM_k_ to overall variance
*rVAR*_*k*_	Relative variances	Relative contribution of PM_k_ to variance produced by trial.
*rSTD*_*k*_	Relative standard deviations	Relative contribution of PM_k_ to movement of trial.
*CUM*_*rEV*_*k*_	Cumulative relative eigenvalues	Cumulative relative contribution of PM_k_ to overall variance.
*CUM*_*rVAR*_*k*_	Cumulative relative variances	Cumulative relative contribution of PM_k_ to variance produced by trial.
*CUM*_*rSTD*_*k*_	Cumulative relative standard deviations	Cumulative relative contribution of PM_k_ to movement in trial.
*RV*_*m*_	Residual variance	Unexplained variance after setting threshold of PM-order *m*.
*N*_*k*_	number of PA zero crossings	Number of interventions of the control system with respect to the movement defined by PM_k_
σ_*k*_	Standard deviation of times between zero crossings	Standard deviation of times between the interventions of the control system with respect to the movement defined by PM_k_

### PCA Validity Considerations

To quantify which ***PC***_***k***_ basis is adequate to describe the group as a whole, a leave-one-out cross-validation can be performed (Diana and Tommasi, 2002; Bro et al., 2008; Camacho and Ferrer, 2012). Therefore, the ***PC***_***k***_ are computed several times, while omitting one trial each time. The changes between the used ***PC***_***k***_ and the newly obtained $P{C^{\prime }}_{k}$ can be quantified as angles and serve as a PM-inclusion criterion (Federolf, 2016; Haid and Federolf, 2018; Haid et al., 2018).

## Results—The PManalyzer Software

### The Interface

**Figure 1:**
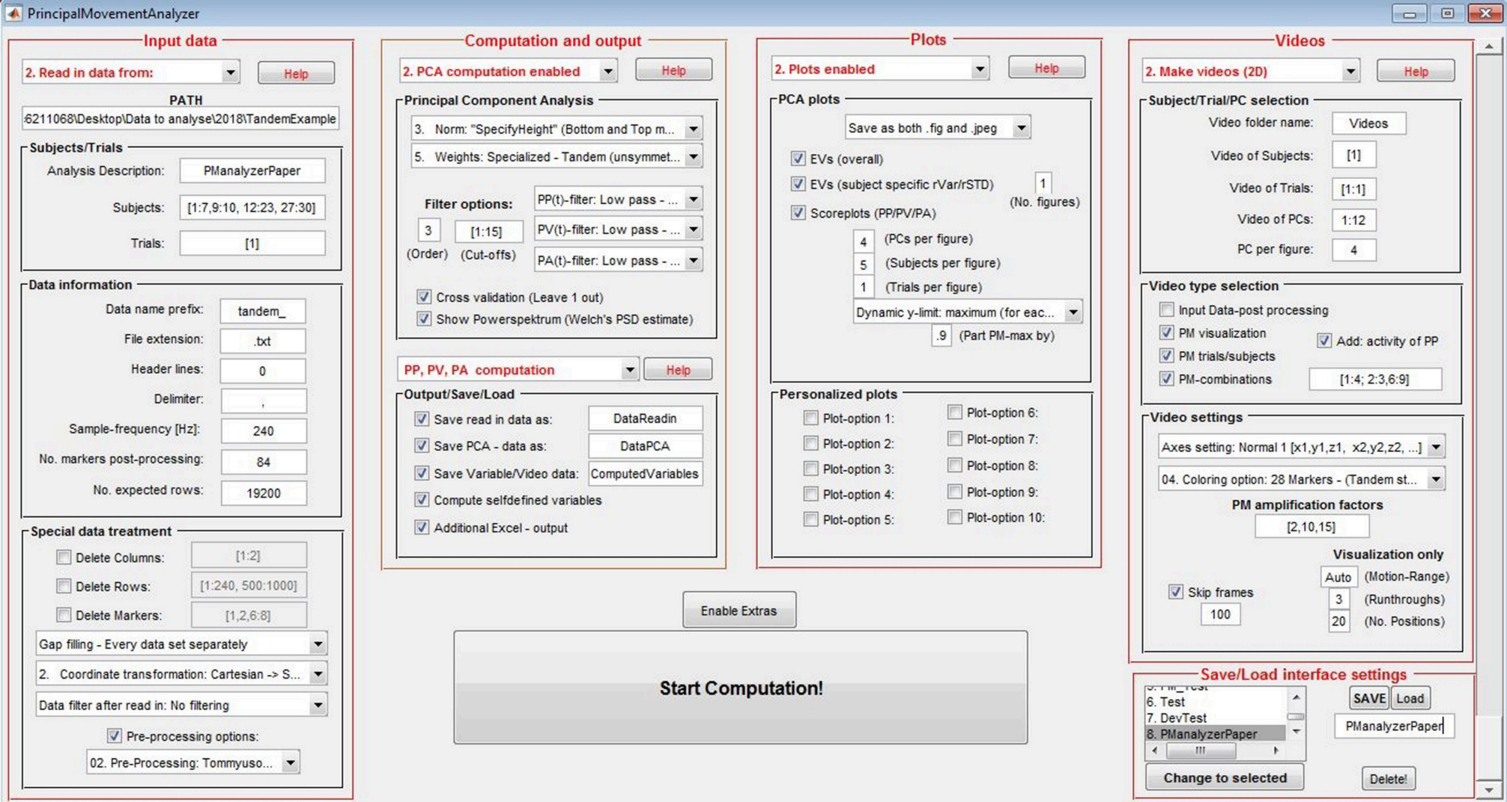
General user interface (GUI) of the PManalyzer. The input settings shown here were used for the computation of the example discussed in the current paper.

**Figure 2:**
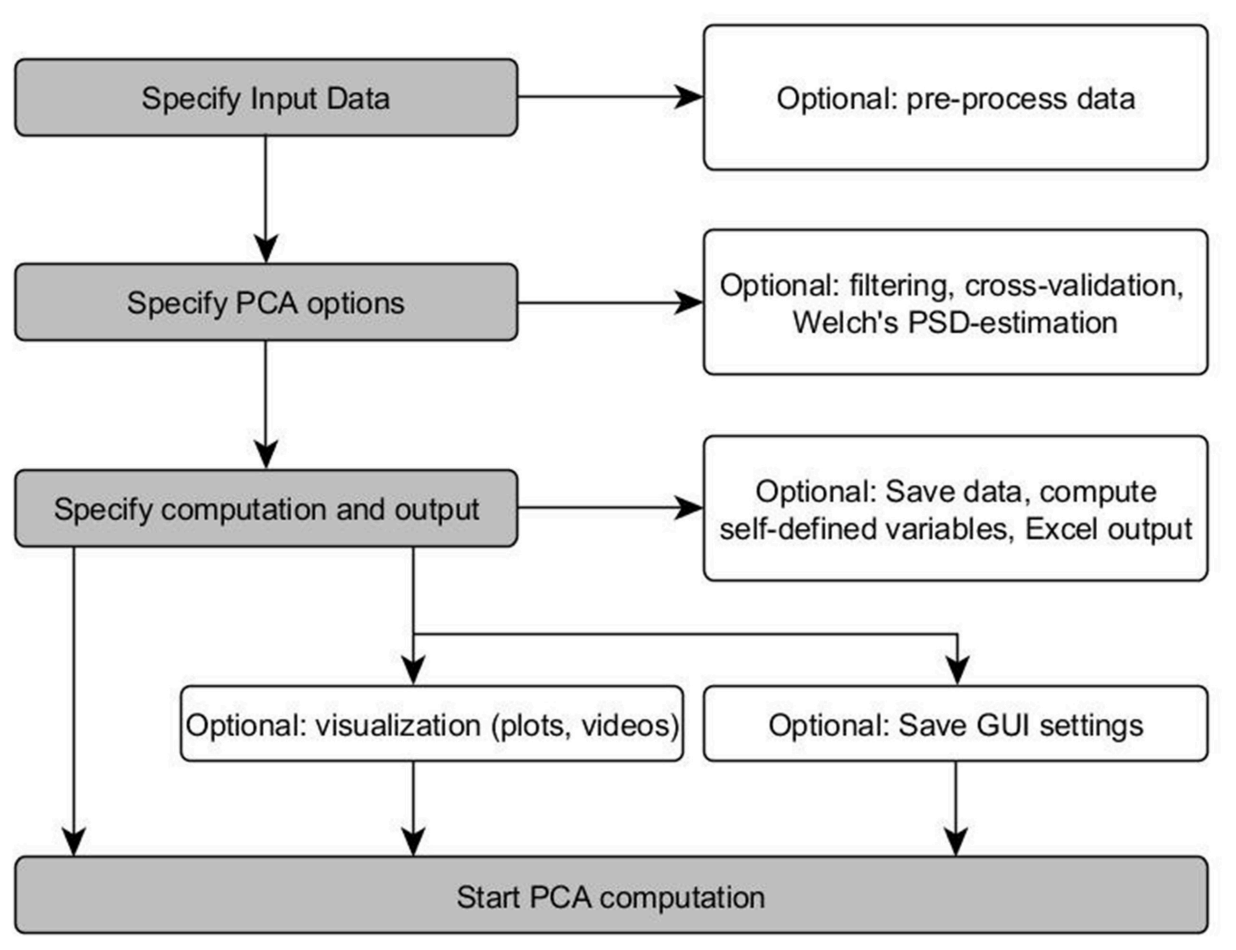
Block-scheme of the PManalyzer computation options. Gray fields describe essential parameter selections. White field represent optional GUI features (Welch's PSD-estimation can be used to estimate the power spectral density of data and to determine a plausible cut-off frequency).

As depicted in Figure 1, the PManalyzer interface is organized into five main panels with red margin and font: 1. “Input data,” 2. “Computation and output,” 3. “Plots,” 4. “Videos” and 5. “Save/Load interface settings.” Following the subpanels one by one allows the user to move through the conventional steps for a PCA applied to kinematic data as described in the section Materials and methods. The block scheme in Figure 2 visualizes the steps of the parameter selection when using the PManalyzer.

Once the computational options are selected, the user can save interface settings and reload them later if needed. To improve efficiency when repeating calculation steps, computed data can also be saved, and loaded. The compatibility of the computing vs. loading vs. disabling options is regulated over the interface to avoid the selection of incompatible features.

Note: The interface was created with the “guide” tool in “MATLAB 2015a” in “Windows 10” on a screen with a 1,920 × 1,080 resolution. Both “Units” and “FontUnits” were set to “normalized” with respect to screen size. For other software or hardware configurations (for example on Mac books) some adaptations may be necessary. Also, some of the plotting features may produce errors if the PManalyzer is run on earlier versions of MATLAB^TM^.

### Code Structure and Computation

The source code is built upon the structure of the user interface and kinematic PCA described in the methods. To monitor the code activity a text describing the current computational step is printed in the output-console. Furthermore, the code is documented by comments to identify the task of each code section and help identifying important computational variables and their respective role in the code. Despite the self-regulating interface, it is possible to select options that do not match the data. The code has implemented fail-safes to identify obvious selection errors and forward them to the user, e.g., when users choose to make video files of data that was not read in.

Functions containing computational options meant for the user to customize (pre-processing, coordinate transformations, normalizations, weighting, variables on PM time-series, video-coloring and creating additional plots) are contained in the PManalyzer subfolder “FunctionsToEdit.” Users can follow the descriptions and the examples provided inside each function to implement their new options. When starting, the GUI automatically loads all functions contained in subfolders and updates the interface with the available options.

### Application Example

In this section, an example computation will be presented to highlight the flexibility of the software. Then, a standard PCA-analysis procedure is outlined. The input is a data subset taken from a previously published tandem stance study that served as template for the PManalyzer (Haid et al., 2018).

#### Computational Parameters and Modifications

For the sample tandem-stance data the first two columns containing time-frames and the headers were deleted. Then gap-filling (Gløersen and Federolf, 2016) was performed on each data set if needed, and a pre-processing option was created that mirrors specific data to make it comparable (unsymmetrical markers were deleted and data with left foot in front was mirrored). The data was then centered, weighted to standard human mass distribution (Defense Technical Information Center, 1988) and normalized with the height of the participants. We also filtered the data with a low-pass filter of 7 Hz, since Fourier analysis suggested signal power up to this frequency. As this example shows, standard pre-processing options can be performed on all of the data by simple parameter selection.

Another interesting pre-processing option that is rarely taken advantage of in kinematic PCA-research is a coordinate transformation. The PManalyzer has two types of coordinate transformations pre-implemented (spherical and cylindrical). Hence, we recomputed the analysis twice using the same parameter selection as described above, but transforming the data either into spherical or cylindrical coordinates, respectively.

Moreover, we selected several of the standard plotting options for the standard PCA variables (*rEV*_*k*_, *rSTD*_*k*_, *rVAR*_*k*_, ***PP***, ***PV***, ***PA***). Further variables such as *N*_*k*_ or σ_*k*_ (Haid et al., 2018; Promsri et al., 2018a) can be computed by selecting “Compute selfdefined variables” and can either be analyzed via Excel output or plotted by defining plots in the function *personalizedPlots.m*. In addition, we selected several video options (2D, 3D, and three different coloring choices. The Supplementary Files contain a summary of the important results of these computations, which we will discuss in the following section.

#### PCA Results

**Figure 3:**
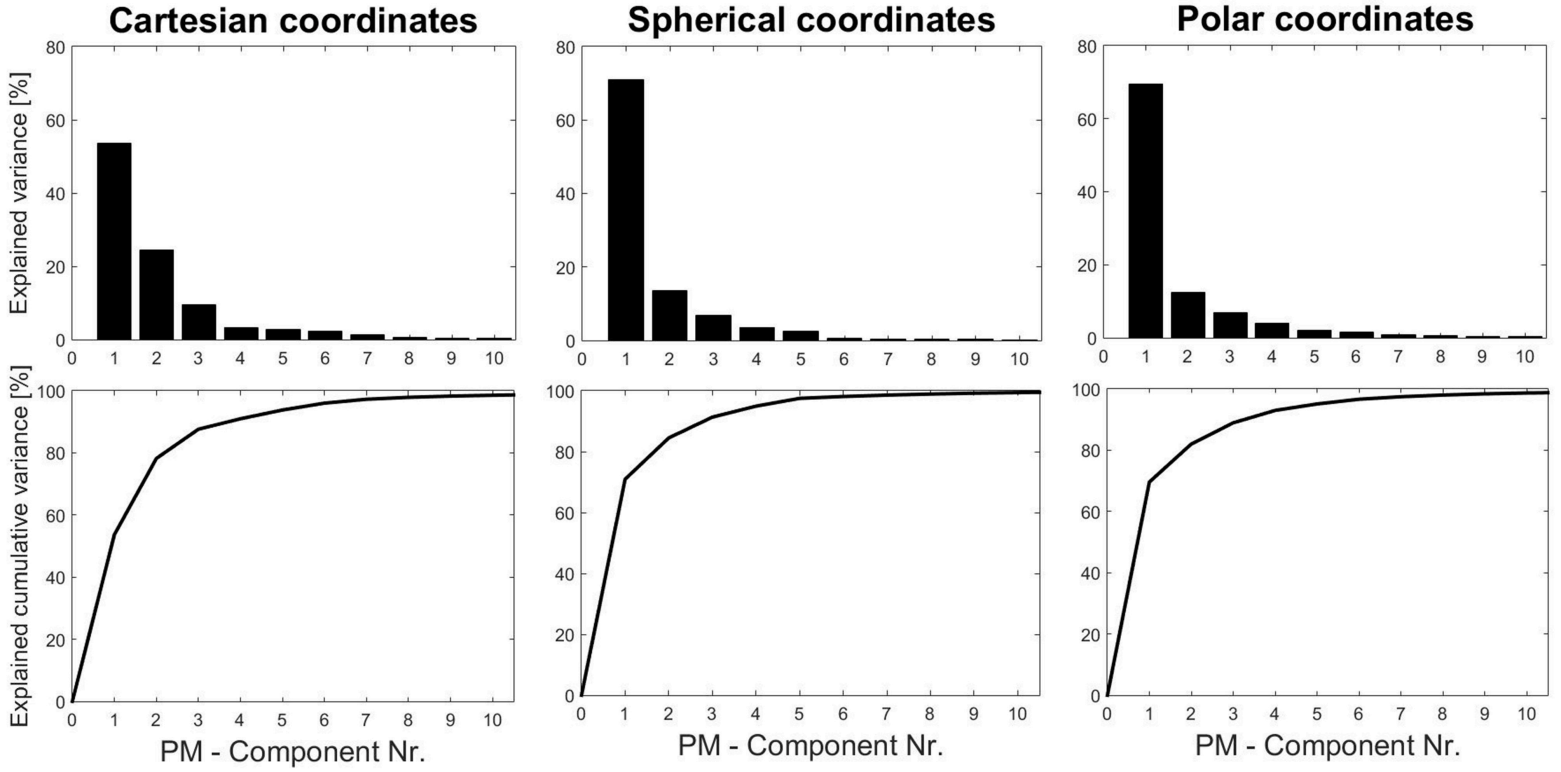
Eigenvalue and cumulative eigenvalue spectra obtained from three coordinate systems (standard kinematic PCA applications use Cartesian-coordinate systems). To explain roughly 98% of the variance it takes 9 PMs using Cartesian, 8 PMs using polar and 6 PMs using spherical coordinates.

As a common first step, the overall eigenvalues were analyzed to see how much overall variance can be explained by the components (individually or cumulatively). These results (Figure 3) show that using spherical or cylindrical coordinate transformations would allow to explain more variance with fewer components. Therefore, we chose to continue the analysis with the results obtained by using spherical coordinates.

As a next step, the PM-movies can be used to describe the movement components to form a better understanding of the extracted movements (compare “ColoringNone_2D_PM1-5_vis.mp4”). It is often helpful to implement specific coloring options (compare “Coloring1_2D_PM1-5_vis.mp4” and “Coloring1_2D_PM1-5_vis.mp4”). For this sample data, the first principal movement resembled an anteroposterior ankle sway. The second PM resembled an upper body retraction accompanied by front knee flexion, etc. The amplification factors displayed in the titles can be adjusted individually for every PM_k_. This is helpful when identifying movements of different magnitudes.

**Figure 4:**
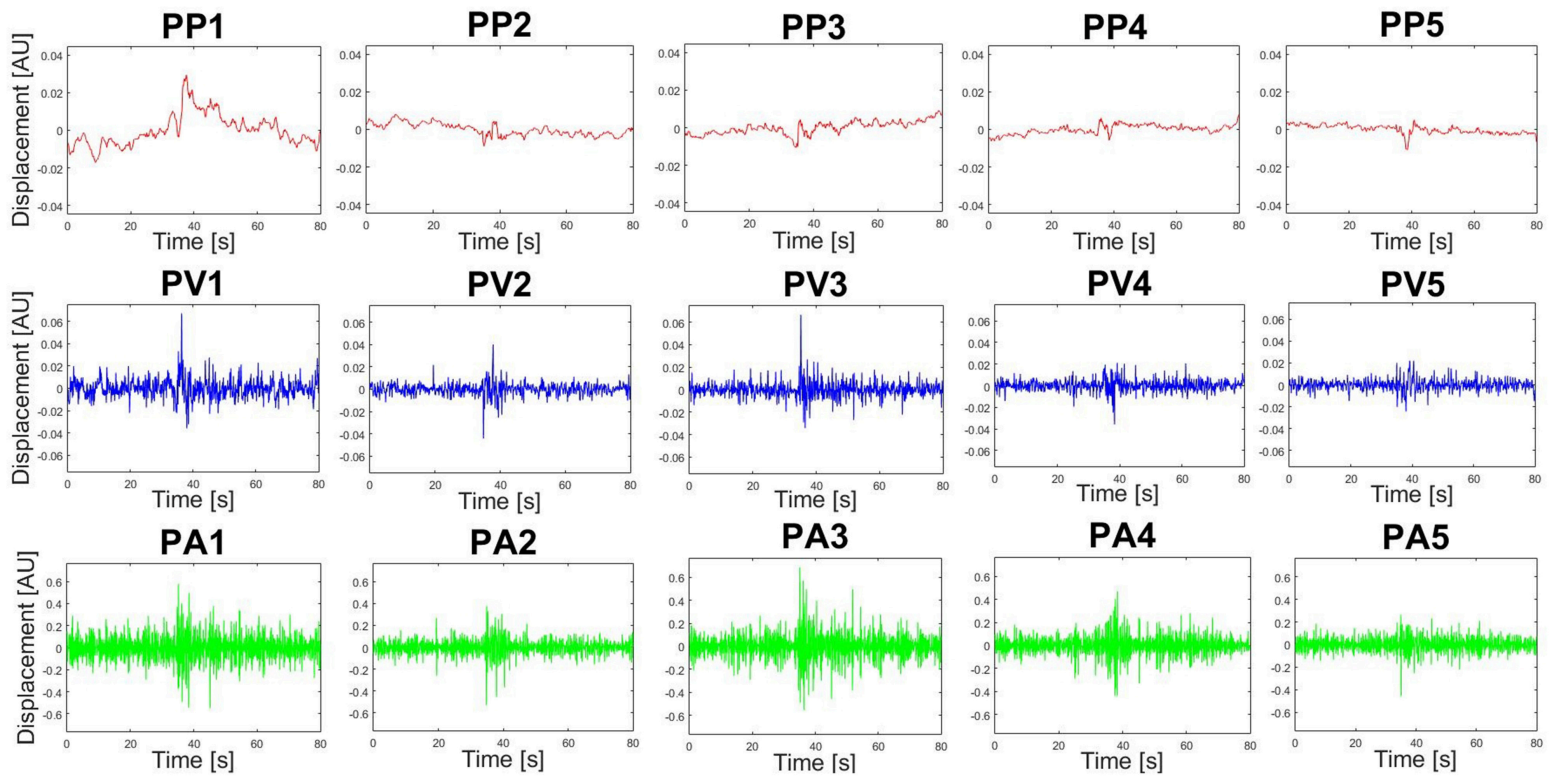
Exemplary PP-, PV- and PA-time-series produced by the PManalyser. This specific data was recorded from a subject performing a tandem stance balance trial. The number of trials, subjects and PMs displayed per figure can be selected in the interface. Units are arbitrary (AU), since they represent a combined motion of all markers and may depend on pre-processing options.

**Figure 5:**
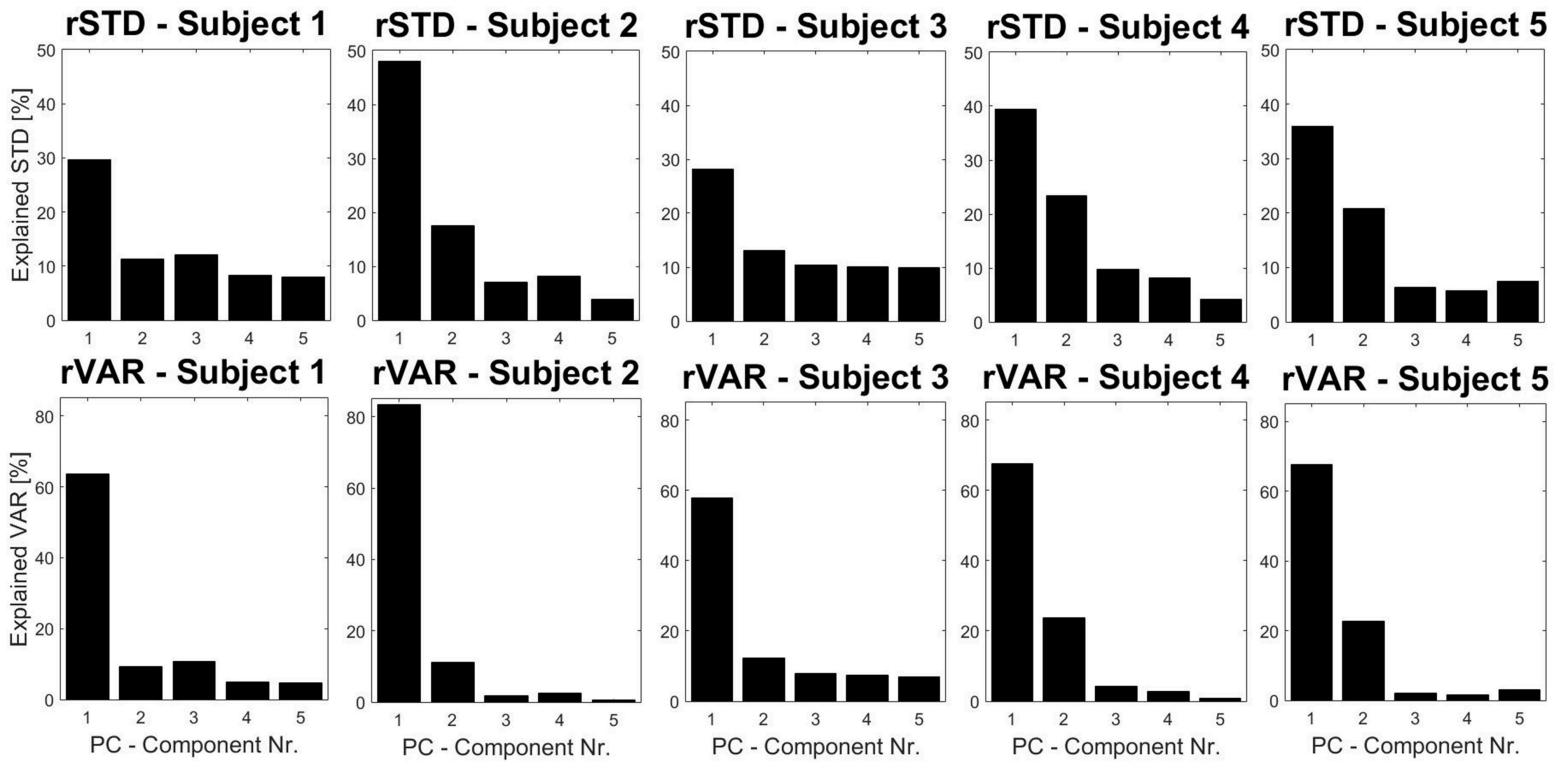
Subject specific relative variances and standard deviations (rSTD and rVAR) for five subjects performing a tandem stance, using spherical coordinates. These eigenvalues are useful to compare the coordinative structure of a movement. In a similar fashion to Figure 3 the cumulative versions of the variables can also be plotted with the software.

Furthermore, PM time-series plots show the execution of the individual trials with respect to the extracted movements (see Figure 4) and the PP activity over time can also be displayed in the video option (“Subject1_2D_PM-5.mp4” and “Subject1_3D_PM-5.mp4”). Both can be useful developing hypotheses related to the dynamics of PMs or their interplay. Users may define any sort of variable in the function *optionsVariablesComp.m*. These variables can then be computed on ***PP***_***k***_ -, ***PV***_***k***_- and ***PA***_***k***_-time-series, thus describing specific aspects of movement components that were not a priori defined, but play an important role producing the observed variance. As an example, we plotted the trial specific relative variances *rVAR*_*k*_ and standard deviations *rSTD*_*k*_ that have been very useful when comparing movement structures amongst various groups (Federolf, 2016; Haid et al., 2018; Promsri et al., 2018a). In the current example it can be observed that while the overall movement of subject 2 is dominated by anteroposterior ankle sway, subject 3 has a more balanced movement structure, where several movements contribute effectively (Figure 5).

## Discussion

### Application of PCA-Variables

In human movement analyses, one of the most important steps is the reduction of the numerous degrees of freedom. Several approaches have been proposed in order to reduce the DOF while capturing the most important dynamics of human movements. For example, in static balance research, one of the most common approaches is to quantify the center of pressure movement, reducing the complex whole-body kinematics to the resultant point where the vertical ground reaction forces act. Indeed, COP based variables proved to be effective at distinguishing different pathological groups and different balancing conditions. However, literature findings are inconsistent and some interpretations are controversial. For example, COP-irregularity has been interpreted as a sign of very active and effective postural control (Cavanaugh et al., 2006; Donker et al., 2007; Haran and Keshner, 2008; Stins et al., 2009; De Beaumont et al., 2011), but also as a sign of a disordered and less effective control (Donker et al., 2007; Stins et al., 2009; Borg and Laxåback, 2010; Gao et al., 2011).

Reducing the DOF via PCA has helped to address some of the inconsistencies in COP literature. As a first step it was shown that the information contained in the COP-excursion should also be contained in PCA variables, since the COP-trajectories can be calculated from the ***PP***_***k***_ and ***PA***_***k***_ time series (Federolf, 2016). Then, follow-up research found that COP-irregularity correlates with both the mechanical complexity of the movement, as quantified by the movement structure *rSTD*_*k*_, and the irregularity of the neuromuscular control as quantified by ***PP***_***k***_-irregularity (Haid and Federolf, 2018). Hence, these findings suggest that COP-irregularity depends on more than one interacting phenomenon, possibly explaining some of the controversial results.

As another example, in research areas that involve postural control and motor control theories, e.g., neurosciences, distinguishing movement strategies can be of great importance. For example, the minimal intervention principle MIP, as discussed in the context of the optimal feedback control theory (Todorov and Jordan, 2002), states that postural control focuses on task relevant movements, while allowing variability in redundant ones. Furthermore, evidence suggests that ankle, knee and hip strategies dominate the whole-body kinematics of balancing tasks (Gage et al., 2004; Kuznetsov and Riley, 2012). In addition, coherence analyses of respective joint angles (Kilby et al., 2015; Masumoto and Inui, 2015) and muscle-EMGs (Alfuth and Gomoll, 2018; Pollock et al., 2019) suggest that these strategies are coordinated (Huisinga et al., 2017; Shahvarpour et al., 2018). Nevertheless, further evidence suggests that when modeling the dynamics according to these segment interactions (Oliveira et al., 2017; McNair et al., 2018), the models seem unable to explain the full extent of the movement dynamics (Hume et al., 2019). Hence, since these studies depend on only a few pre-selected muscles and DOF they might be limited when testing hypotheses related to the MIP.

The advantage of the PCA approach is that the extracted principal movement components are inherent in the data. They represent coordinated marker movements that generate quantifiable amounts of the overall variance produced by the analyzed movement. This allows categorizing them with respect to their relative contribution to the overall movement and to determine a movement's composition of PM, i.e. the movement structure (*rSTD*). Furthermore, the respective PM-time-series can be used to quantify novel aspects of postural control, such as how tight a movement is controlled (how often the control system intervenes (*N*_*k*_) and how variable the control (σ_*k*_) of the respective PM_k_ is). As an example, in accordance with the MIP the tandem stance study mentioned in the results of this paper (Haid et al., 2018) found that aging effects emerged in the movement structure and control of specific, task relevant components, but did not affect other movement components. In detail, the movement component with the least base of support exhibited less relative contribution and tighter control in the younger age group. Also leg dominance was studied in a similar fashion (Promsri et al., 2018b) revealing differing movement control characteristics in different movement components.

In addition, the PCA variables were used in several studies with clinical purposes, or for fundamental research. Specifically, they were helpful to classify gait patterns that are a result of spastic diplegia (Zago et al., 2017c), affect (Karg et al., 2010), gender or age (Troje, 2002; Verrel et al., 2009; Eskofier et al., 2013), or shoe material (Maurer et al., 2012; von Tscharner et al., 2013). Principal movements were also calculated as pre-processing step in research on work-related musculoskeletal disorders that aimed at characterizing the variability and the local dynamic stability of the movements (Longo et al., 2018a,b). The PM calculation allowed distinguishing cycle-to-cycle variability from changes in the overall postural configuration—a prerequisite for the calculation of non-linear variables such as the largest Lyapunov exponent in this context. In sports, coordinative strategies were studied, by identifying and quantifying PCA-eigenvectors, eigenvalues and score time-series, for example in alpine skiing (Federolf et al., 2014), cross-country skiing (Gløersen et al., 2017), Karate (Zago et al., 2017a), dancing (Masurelle et al., 2013), cycling (Moore et al., 2011), diving (Young and Reinkensmeyer, 2014), and race-walking (Donà et al., 2009).

In summary, literature suggests that kinematic PCA can be an effective tool to study pathological conditions or sport performance, and to address unsolved problems of motor control theories such as the minimal intervention principle. The basic code structure of the PManalyzer was originally developed for the tandem stance study (Haid et al., 2018). Later, the code was further developed to be applicable in a wider range of static balancing tasks. However, as discussed in the following section, it is also modifiable to be used in other application areas.

### Computational Features and Advantages of the Software

The main purpose of the PManalyzer software was to make PCA computations more easily accessible for users, particularly for users less familiar with programming or with the mathematical background of PCA applications. The PManalyzer offers the broad spectrum of available computational options and the large variety of easily customizable visualization options. It also allows a user to perform pre-processing steps like PCA-based gap-filling (Gløersen and Federolf, 2016), deleting markers, columns or rows, or to integrate any other self-defined data pre-processing steps. Additionally, the PManalyzer can transform data from a Cartesian into a spherical or polar coordinate system. Users with more advanced mathematical knowledge can implement further coordinate transformations. Moreover, a number of pre-defined normalization options are available, of which two have been validated (mean Euclidean distance and height) through previous research (Federolf P. et al., 2013; Zago et al., 2017b; Haid et al., 2018; Promsri et al., 2018a), while others (such as maximum movement range in x, y, or z direction) have yet to be explored. Also the weighting options for the standard 39 and 37 (no fingers) plug-in gate marker systems are pre-implemented, as well as the specialized 28 marker system (only symmetric markers) used in the tandem stance study of the results (Haid et al., 2018).

Furthermore, new variables can easily be implemented to be computed on all PM-time-series. If selected, they will be saved with the other variables on the ***PP*-**, ***PV*-** and ***PA*-**time-series (*rVAR*, *rSTD*, *N*, σ, RMS, mean, standard deviation, amongst others). For users not familiar with Matlab programming, the results of all computed variables can be exported to an Excel spread sheet. Moreover, users can create customized plots that are directly integrated into the interface. Finally, any video coloring option can be added to the software without extensive Matlab skills, saving programming time and effort.

To validate the obtained basis ***PC_k_***, a leave one out cross-validation has been implemented that produces a figure displaying the angle-changes as described in section PCA validity considerations. Furthermore, a figure containing a Welch's power spectral density estimate can be created to help determine a suitable filtering frequency. Moreover, specifying a vector of cut-off frequencies will run the selected PCA-computations consecutively with different filtering cut-off frequencies and saving the results in separate folders. This is particularly useful, in order to conduct a frequency analysis to ensure that statistical results are stable for various cut-off frequencies (Haid and Federolf, 2018; Haid et al., 2018; Promsri et al., 2018a).

### Limitations and Future Research Potential

When it comes to effectively applying kinematic PCA and to establishing reliable norm values for a clinical and sports related environment, several factors should be considered. First, kinematic PCA is only one of many interesting feature extraction algorithms. For example, independent component analysis (von Tscharner et al., 2013), isometric feature mapping (Blackburn et al., 2003) and linear discriminant analysis (Karg et al., 2010) have been used as kinematic feature extraction tools and shown to outperform PCA in specific situations. Hence, there is tremendous potential for systematic research into the advantages and disadvantages of PCA compared to several other feature extraction techniques (Van Der Maaten et al., 2009).

Second, in order to establish norm values it is essential to define standard procedures. Hence, marker systems, pre-processing options, normalization and weighting, and coordinate transformations must be explored and standardized for different types of movements. Specifically, coordinate transformations are an interesting, yet relatively unexplored feature in kinematic PCA. As an example, the tandem-stance study analyzed nine different ankle, knee, upper body and head strategies, explaining 98% of the overall variance. The results in this study show that only 6 PMs would be necessary to achieve the same accuracy, if spherical coordinates were used. Furthermore, also moving coordinate systems offer unexplored potential. The example of alpine skiing technique analysis (Federolf et al., 2014) shows that body-positioning-dependent coordinate systems can help focus the analysis by neglecting movements with respect to specified planes. A similar, implemented pre-processing feature in the PManalyzer is the pre-processing option that moves the coordinate system into the center of mass, which can be used to avoid body displacements being represented as PMs.

Third, PCA based variables described in this study have been applied successfully to quantify movement coordination and complexity (*rEV*, *rVAR*, *rSTD*, and *RV*), and movement control (*N*, σ, ***PP***-irregularity), amongst others. However, especially the variables of movement control computed on the ***PA***-time-series (*N*, σ) react sensitively to the quality of kinematic data and filtering settings, due to double differentiation of the data. Nevertheless, a frequency analysis of the variables of movement control indicated that the underlying effects are robust to changes in filtering frequency and not random artifacts (Haid and Federolf, 2018; Haid et al., 2018; Promsri et al., 2018a). Hence, it should be possible to use PCA variables to establish objective norm values that describe movement performance. However, follow-up research is needed to further validate existing variables and possible to develop new ones.

Finally, the extracted principal movements must be carefully interpreted. Each PM is defined by one linear movement of each marker. Since real whole-body movements are usually not linear, individual PMs can only approximate real movements, at best. However, some of the PMs obtained from movements with small amplitudes, such bipedal static balancing tasks (Federolf, 2016; Haid et al., 2018), seem to be realistic approximations of movement strategies that were already described in the literature, such as ankle sway and hip-strategies (Gage et al., 2004; Kuznetsov and Riley, 2012; Kilby et al., 2015). Others, such as certain upper body strategies have not been described in literature but seem realistic in the author's eyes. Furthermore, non-linear movements with higher amplitudes would require at least two or more PMs to be approximated in a realistic way. In theory, this limitation could be overcome with specialized non-linear coordinate transformations or other feature extraction techniques. At the moment, evidence suggests that the PMs of higher amplitude movements describe interesting features that allow group classifications, e.g., gait recognition (Troje, 2002; Verrel et al., 2009; Karg et al., 2010) or sport performance (Donà et al., 2009; Federolf et al., 2014; Young and Reinkensmeyer, 2014). However, further research is needed to link specific linear PM-combinations to realistic non-linear movements.

In terms of the PManalyzer, some of the GUI options, for example weighing markers according to the segment masses they represent, depend on the input data (number and distribution of markers) and the type of movement analyzed. A flexible usage requires the user to define these options for non-standardized input data, since, specifically for these options, the software relies on pre-implemented options rather than on software recognition. However, only basic, easily acquirable Matlab knowledge is needed to follow the templates in the editable functions and to perform such changes in the according functions. Furthermore, despite beta testing, bugs can never be excluded. Nevertheless, we are confident that the software works well, as it has been tested on various data sets (Haid and Federolf, 2018; Haid et al., 2018; Longo et al., 2018a,b, 2019; Promsri et al., 2018a,b), yielding the expected results. We encourage the community to report possible improvements to the authors.

## Conclusions

We presented the PManalyzer, a software tool that is meant as a basis code for applying PCA in the analysis of human movement and its sensorimotor control. We hope this will encourage colleagues to more often apply PCA in their movement control related research. The computational options are not meant to be complete, but rather to enable easy software modifications to assist future users in the development of specialized applications.

## Ethics Statement

The example data in section Discussion was taken from a study that was conducted in agreement with the Declaration of Helsinki, in particular, an institutionalized ethics review board had approved the study design and informed written consent was obtained from all volunteers prior to any measurements.

## Author Contributions

TH developed and implemented the PManalyzer, and wrote the first draft of the paper, the quick start guide and the user's manual, constantly updating the software and the manuscripts. MZ validated the software's computational steps, by comparing the PCA outputs to previously published results, and repeatedly revised both the paper and the starter guide. AP contributed by challenging several software-updates as they became available and helped eliminating numerous update-bugs. She also gave valuable insights on how to improve the software's usability and revised the starter guide. A-CD tested the functionality and adaptability of the software, improving code-documentation and contributed to the manuscript and the quick start guide. PF contributed as mentor and PCA-teacher, which lead to the PManalyzer software project. For this paper he was also deeply involved in the writing-process, improving its structure, and wording. Moreover, his regular and critical software evaluations drastically improved the PManalyzer.

### Conflict of Interest Statement

The authors declare that the research was conducted in the absence of any commercial or financial relationships that could be construed as a potential conflict of interest.

## Footnotes

^1^The default order in the software is centering, then weighting and finally normalizing the individual data sets. Depending on the pre-processing selection, the order might influence the results.

^2^In literature the “relative eigenvalues” rEV_k_ are sometimes referred to as EV_k_ out of simplicity. In addition, the term “relative eigenvalues” may refer to the “trial specific relative variances” as described in the following section. In this manuscript we aim at consistency.

^4^Residual variances can also be computed for the overall contributions by substituting the rVARk with the rEVk.

## Supplementary Material

The Supplementary Material for this article can be found online at: https://www.frontiersin.org/articles/10.3389/fninf.2019.00024/full#supplementary-material Supplementary materials (the PManalyzer software, documentation, data examples and video files) can be found online at: https://www.uibk.ac.at/isw/forschung/neurophysiologie-bewegungswissenschaft/software.html.

The website contains the most recent, updated versions of the software, a quick start guide “GettingStarted_PManalyzer.docx” and a user's manual (“UsersManual_PManalyzer.pdf”).

## References

[B1] Alfuth M. Gomoll M. (2018). Electromyographic analysis of balance exercises in single-leg stance using different instability modalities of the forefoot and rearfoot. Phys. Ther. Sport Off. J. Assoc. Chart. Physiother. Sports Med. 31, 75–82. 10.1016/j.ptsp.2018.01.002 29573984

[B2] Bernstein N. (1967). The Co-Ordination and Regulation of Movements. Oxford: Pergamon-Press.

[B3] Blackburn J. T. Riemann B. L. Myers J. B. Lephart S. M. (2003). Kinematic analysis of the hip and trunk during bilateral stance on firm, foam, and multiaxial support surfaces. Clin. Biomech. 18, 655–661. 10.1016/S0268-0033(03)00091-3 12880713

[B4] Borg F. G. Laxåback G. (2010). Entropy of balance–some recent results. J. Neuroeng. Rehabil. 7:38. 10.1186/1743-0003-7-38 20670457 PMC2923165

[B5] Boström K. J. Dirksen T. Zentgraf K. Wagner H. (2018). The contribution of upper body movements to dynamic balance regulation during challenged locomotion. Front. Hum. Neurosci. 12:8. 10.3389/fnhum.2018.00008 29434544 PMC5790866

[B6] Bro R. Kjeldahl K. Smilde A. K. Kiers H. A. L. (2008). Cross-validation of component models: a critical look at current methods. Anal. Bioanal. Chem. 390, 1241–1251. 10.1007/s00216-007-1790-1 18214448

[B7] Camacho J. Ferrer A. (2012). Cross-validation in PCA models with the element-wise k-fold (ekf) algorithm: theoretical aspects. J. Chemom. 26, 361–373. 10.1002/cem.2440

[B8] Cavanaugh J. T. Guskiewicz K. M. Giuliani C. Marshall S. Mercer V. S. Stergiou N. (2006). Recovery of postural control after cerebral concussion: new insights using approximate entropy. J. Athl. Train. 41, 305–313. 17043699 PMC1569549

[B9] Daffertshofer A. Lamoth C. J. C. Meijer O. G. Beek P. J. (2004). PCA in studying coordination and variability: a tutorial. Clin. Biomech. 19, 415–428. 10.1016/j.clinbiomech.2004.01.005 15109763

[B10] De Beaumont L. Mongeon D. Tremblay S. Messier J. Prince F. Leclerc S.. (2011). Persistent motor system abnormalities in formerly concussed athletes. J. Athl. Train. 46, 234–240. 10.4085/1062-6050-46.3.234 21669091 PMC3419550

[B11] de Leva P. (1996). Adjustments to Zatsiorsky-Seluyanov's segment inertia parameters. J. Biomech. 29, 1223–1230. 8872282 10.1016/0021-9290(95)00178-6

[B12] Defense Technical Information Center (1988). DTIC ADA304353: Anthropometry and Mass Distribution for Human Analogues, Volume I. Military Male Aviators. Available online at: http://archive.org/details/DTIC_ADA304353 (accessed April 25, 2018).

[B13] Diana G. Tommasi C. (2002). Cross-validation methods in principal component analysis: a comparison. Stat. Methods Appl. 11, 71–82. 10.1007/BF02511446

[B14] Donà G. Preatoni E. Cobelli C. Rodano R. Harrison A. J. (2009). Application of functional principal component analysis in race walking: an emerging methodology. Sports Biomech. 8, 284–301. 10.1080/14763140903414425 20169759

[B15] Donker S. F. Roerdink M. Greven A. J. Beek P. J. (2007). Regularity of center-of-pressure trajectories depends on the amount of attention invested in postural control. Exp. Brain Res. 181, 1–11. 10.1007/s00221-007-0905-4 17401553 PMC1914290

[B16] Eskofier B. M. Federolf P. Kugler P. F. Nigg B. M. (2013). Marker-based classification of young-elderly gait pattern differences via direct PCA feature extraction and SVMs. Comput. Methods Biomech. Biomed. Eng. 16, 435–442. 10.1080/10255842.2011.624515 22149087

[B17] Federolf P. Reid R. Gilgien M. Haugen P. Smith G. (2014). The application of principal component analysis to quantify technique in sports. Scand. J. Med. Sci. Sports 24, 491–499. 10.1111/j.1600-0838.2012.01455.x 22436088

[B18] Federolf P. Roos L. Nigg B. (2012). The effect of footwear on postural control in bipedal quiet stance. Footwear Sci. 4, 115–122. 10.1080/19424280.2012.666270

[B19] Federolf P. Roos L. Nigg B. M. (2013). Analysis of the multi-segmental postural movement strategies utilized in bipedal, tandem and one-leg stance as quantified by a principal component decomposition of marker coordinates. J. Biomech. 46, 2626–2633. 10.1016/j.jbiomech.2013.08.008 24021753

[B20] Federolf P. A. (2016). A novel approach to study human posture control: “Principal movements” obtained from a principal component analysis of kinematic marker data. J. Biomech. 49, 364–370. 10.1016/j.jbiomech.2015.12.030 26768228

[B21] Federolf P. A. Boyer K. A. Andriacchi T. P. (2013). Application of principal component analysis in clinical gait research: identification of systematic differences between healthy and medial knee-osteoarthritic gait. J. Biomech. 46, 2173–2178. 10.1016/j.jbiomech.2013.06.032 23910389

[B22] Figueroa P. J. Leite N. J. Barros R. M. L. (2003). A flexible software for tracking of markers used in human motion analysis. Comput. Methods Programs Biomed. 72, 155–165. 10.1016/S0169-2607(02)00122-0 12941519

[B23] Fino P. C. Nussbaum M. A. Brolinson P. G. (2016). Decreased high-frequency center-of-pressure complexity in recently concussed asymptomatic athletes. Gait Post. 50, 69–74. 10.1016/j.gaitpost.2016.08.026 27580081

[B24] Gage W. H. Winter D. A. Frank J. S. Adkin A. L. (2004). Kinematic and kinetic validity of the inverted pendulum model in quiet standing. Gait Post. 19, 124–132. 10.1016/S0966-6362(03)00037-7 15013500

[B25] Gallagher D. Heymsfield S. B. (1998). Muscle distribution: variations with body weight, gender, and age. Appl. Radiat. Isot. 49, 733–734. 9569594 10.1016/s0969-8043(97)00096-1

[B26] Gao J. Hu J. Buckley T. White K. Hass C. (2011). Shannon and Renyi entropies to classify effects of Mild Traumatic Brain Injury on postural sway. PLoS ONE 6:e24446. 10.1371/journal.pone.0024446 21931720 PMC3170368

[B27] Gløersen Ø. Federolf P. (2016). Predicting missing marker trajectories in human motion data using marker intercorrelations. PLoS ONE 11:e0152616. 10.1371/journal.pone.0152616 27031243 PMC4816448

[B28] Gløersen Ø. Myklebust H. Hallén J. Federolf P. (2017). Technique analysis in elite athletes using principal component analysis. J. Sports Sci. 36, 229–237. 10.1080/02640414.2017.1298826 28287028

[B29] Haid T. Federolf P. (2018). Human postural control: assessment of two alternative interpretations of center of pressure sample entropy through a principal component factorization of whole-body kinematics. Entropy 20:30 10.3390/e20010030 PMC7512231 33265120

[B30] Haid T. H. Doix A.-C. M. Nigg B. M. Federolf P. A. (2018). Age effects in postural control analyzed via a principal component analysis of kinematic data and interpreted in relation to predictions of the optimal feedback control theory. Front. Aging Neurosci. 10:22. 10.3389/fnagi.2018.00022 29459826 PMC5807376

[B31] Han A. Fu A. Cobley S. Sanders R. H. (2018). Effectiveness of exercise intervention on improving fundamental movement skills and motor coordination in overweight/obese children and adolescents: a systematic review. J. Sci. Med. Sport 21, 89–102. 10.1016/j.jsams.2017.07.001 28728887

[B32] Haran F. J. Keshner E. A. (2008). Sensory reweighting as a method of balance training for labyrinthine loss. J. Neurol. Phys. Ther. 32, 186–191. 10.1097/NPT.0b013e31818dee39 19265760 PMC2678881

[B33] Honegger F. Tielkens R. J. M. Allum J. H. J. (2013). Movement strategies and sensory reweighting in tandem stance: differences between trained tightrope walkers and untrained subjects. Neuroscience 254, 285–300. 10.1016/j.neuroscience.2013.09.041 24090964

[B34] Huisinga J. Mancini M. Veys C. Spain R. Horak F. (2017). Coherence analysis of trunk and leg acceleration reveals altered postural sway strategy during standing in persons with multiple sclerosis. Hum. Mov. Sci. 58, 330–336. 10.1016/j.humov.2017.12.009 29277247 PMC6390844

[B35] Hume D. R. Navacchia A. Rullkoetter P. J. Shelburne K. B. (2019). A lower extremity model for muscle-driven simulation of activity using explicit finite element modeling. J. Biomech. 84, 153–160. 10.1016/j.jbiomech.2018.12.040 30630624 PMC6361714

[B36] Karg M. Kuhnlenz K. Buss M. (2010). Recognition of Affect Based on Gait Patterns. IEEE Trans. Syst. Man Cybern. Part B Cybern. 40, 1050–1061. 10.1109/TSMCB.2010.2044040 20350859

[B37] Kilby M. C. Molenaar P. C. M. Newell K. M. (2015). Models of postural control: shared variance in joint and COM motions. PLoS ONE 10:e0126379. 10.1371/journal.pone.0126379 25973896 PMC4431684

[B38] Kuznetsov N. A. Riley M. A. (2012). Effects of breathing on multijoint control of center of mass position during upright stance. J. Mot. Behav. 44, 241–253. 10.1080/00222895.2012.688894 22671566

[B39] Lim Y. H. Partridge K. Girdler S. Morris S. L. (2017). Standing postural control in individuals with autism spectrum disorder: systematic review and meta-analysis. J. Autism Dev. Disord. 47, 2238–2253. 10.1007/s10803-017-3144-y 28508177

[B40] Longo A. Federolf P. Haid T. Meulenbroek R. (2018a). Effects of a cognitive dual task on variability and local dynamic stability in sustained repetitive arm movements using principal component analysis: a pilot study. Exp. Brain Res. 236, 1611–1619. 10.1007/s00221-018-5241-3 29589078 PMC5982455

[B41] Longo A. Haid T. Meulenbroek R. Federolf P. (2019). Biomechanics in posture space: properties and relevance of principal accelerations for characterizing movement control. J. Biomech. 82, 397–403. 10.1016/j.jbiomech.2018.11.031 30527635

[B42] Longo A. Meulenbroek R. Haid T. Federolf P. (2018b). Postural reconfiguration and cycle-to-cycle variability in patients with work-related musculoskeletal disorders compared to healthy controls and in relation to pain emerging during a repetitive movement task. Clin. Biomech. 54, 103–110. 10.1016/j.clinbiomech.2018.03.004 29574341

[B43] MacRae C. S. Critchley D. Lewis J. S. Shortland A. (2018). Comparison of standing postural control and gait parameters in people with and without chronic low back pain: a cross-sectional case-control study. BMJ Open Sport Exerc. Med. 4:e000286. 10.1136/bmjsem-2017-000286 29387444 PMC5783032

[B44] Masumoto J. Inui N. (2015). Motor control hierarchy in joint action that involves bimanual force production. J. Neurophysiol. 113, 3736–3743. 10.1152/jn.00313.2015 25904710 PMC4468970

[B45] Masurelle A. Essid S. Richard G. (2013). “Multimodal classification of dance movements using body joint trajectories and step sounds,” in 2013 14th International Workshop on Image Analysis for Multimedia Interactive Services (WIAMIS). Paris, 1–4. 10.1109/WIAMIS.2013.6616151

[B46] Maurer C. Federolf P. von Tscharner V. Stirling L. Nigg B. M. (2012). Discrimination of gender-, speed-, and shoe-dependent movement patterns in runners using full-body kinematics. Gait Post. 36, 40–45. 10.1016/j.gaitpost.2011.12.023 22304784

[B47] McNair P. J. Boocock M. G. Dominick N. D. Kelly R. J. Farrington B. J. Young S. W. (2018). A comparison of walking gait following mechanical and kinematic alignment in total knee joint replacement. J. Arthroplasty 33, 560–564. 10.1016/j.arth.2017.09.031 29054726

[B48] Moore J. K. Kooijman J. D. G. Schwab A. L. Hubbard M. (2011). Rider motion identification during normal bicycling by means of principal component analysis. Multibody Syst. Dyn. 25, 225–244. 10.1007/s11044-010-9225-8

[B49] Nikaido Y. Akisue T. Kajimoto Y. Tucker A. Kawami Y. Urakami H.. (2018). Postural instability differences between idiopathic normal pressure hydrocephalus and Parkinson's disease. Clin. Neurol. Neurosurg. 165, 103–107. 10.1016/j.clineuro.2018.01.012 29331870

[B50] Oliveira A. S. Silva P. B. Lund M. E. Farina D. Kersting U. G. (2017). Balance training enhances motor coordination during a perturbed sidestep cutting task. J. Orthop. Sports Phys. Ther. 47, 853–862. 10.2519/jospt.2017.6980 28944715

[B51] Pollock C. L. Hunt M. A. Vieira T. M. Gallina A. Ivanova T. D. Garland S. J. (2019). Challenging standing balance reduces the asymmetry of motor control of postural sway poststroke. Motor Control. 1–17. 10.1123/mc.2017-0098 30599808

[B52] Promsri A. Haid T. Federolf A. P. (2018a). How does lower limb dominance influence postural control movements during single leg stance? Hum. Mov. Sci. 58, 165–174. 10.1016/j.humov.2018.02.003 29448161

[B53] Promsri A. Haid T. Werner I. Federolf P. (2018b). Influence of lower-limb dominance on coordinative movement structures observed during single-leg balancing on a multiaxial unstable surface. Gait Posture. 65, 60–61. 10.1016/j.gaitpost.2018.06.047

[B54] Sadeghi H. Allard P. Duhaime M. (1997). Functional gait asymmetry in able-bodied subjects. Hum. Mov. Sci. 16, 243–258. 10.1016/S0167-9457(96)00054-1

[B55] Shahvarpour A. Gagnon D. Preuss R. Henry S. M. Larivière C. (2018). Trunk postural balance and low back pain: reliability and relationship with clinical changes following a lumbar stabilization exercise program. Gait Post. 61, 375–381. 10.1016/j.gaitpost.2018.02.006 29448220

[B56] Shlens J. (2014). A tutorial on principal component analysis. arXiv [preprint] arXiv:1404.1100

[B57] Stins J. F. Michielsen M. E. Roerdink M. Beek P. J. (2009). Sway regularity reflects attentional involvement in postural control: effects of expertise, vision and cognition. Gait Post. 30, 106–109. 10.1016/j.gaitpost.2009.04.001 19411174

[B58] Todorov E. Jordan M. I. (2002). Optimal feedback control as a theory of motor coordination. Nat. Neurosci. 5, 1226–1235. 10.1038/nn963 12404008

[B59] Troje N. F. (2002). Decomposing biological motion: a framework for analysis and synthesis of human gait patterns. J. Vis. 2, 371–387. 10.1167/2.5.2 12678652

[B60] Van Der Maaten L. Postma E. Van den Herik J. (2009). Dimensionality reduction: a comparative. J. Mach. Learn. Res. 10, 66–71.

[B61] Verrel J. Lövdén M. Schellenbach M. Schaefer S. Lindenberger U. (2009). Interacting effects of cognitive load and adult age on the regularity of whole-body motion during treadmill walking. Psychol. Aging 24, 75–81. 10.1037/a0014272 19290739

[B62] von Tscharner V. Enders H. Maurer C. (2013). Subspace identification and classification of healthy human gait. PLoS ONE 8:e65063. 10.1371/journal.pone.0065063 23861736 PMC3704597

[B63] Wang Z. Molenaar P. C. M. Challis J. H. Jordan K. Newell K. M. (2014). Visual information and multi-joint coordination patterns in one-leg stance. Gait Post. 39, 909–914. 10.1016/j.gaitpost.2013.11.017 24388780

[B64] Yamagata M. Ikezoe T. Kamiya M. Masaki M. Ichihashi N. (2017). Correlation between movement complexity during static standing and balance function in institutionalized older adults. Clin. Interv. Aging 12, 499–503. 10.2147/CIA.S132425 28331301 PMC5352153

[B65] Young C. Reinkensmeyer D. J. (2014). Judging complex movement performances for excellence: a principal components analysis-based technique applied to competitive diving. Hum. Mov. Sci. 36, 107–122. 10.1016/j.humov.2014.05.009 24968369

[B66] Zago M. Codari M. Iaia F. M. Sforza C. (2017a). Multi-segmental movements as a function of experience in karate. J. Sports Sci. 35, 1515–1522. 10.1080/02640414.2016.1223332 27560105

[B67] Zago M. Pacifici I. Lovecchio N. Galli M. Federolf P. A. Sforza C. (2017b). Multi-segmental movement patterns reflect juggling complexity and skill level. Hum. Mov. Sci. 54, 144–153. 10.1016/j.humov.2017.04.013 28499158

[B68] Zago M. Sforza C. Bona A. Cimolin V. Costici P. F. Condoluci C.. (2017c). How multi segmental patterns deviate in spastic diplegia from typical developed. Clin. Biomech. Bristol Avon 48, 103–109. 10.1016/j.clinbiomech.2017.07.016 28806590

